# Supplementary material for: Ameliorating Effects of Vitamin K2 on Dextran Sulfate Sodium-Induced Ulcerative Colitis in Mice
Source: Int J Mol Sci. 2023 Feb 3;24(3):2986. doi: 10.3390/ijms24032986 (PMC9917520; doi:10.3390/ijms24032986)
Supplement: Supplementary file 1 [file ijms-24-02986-s001.zip › ijms-2154602-supplementary.pdf]

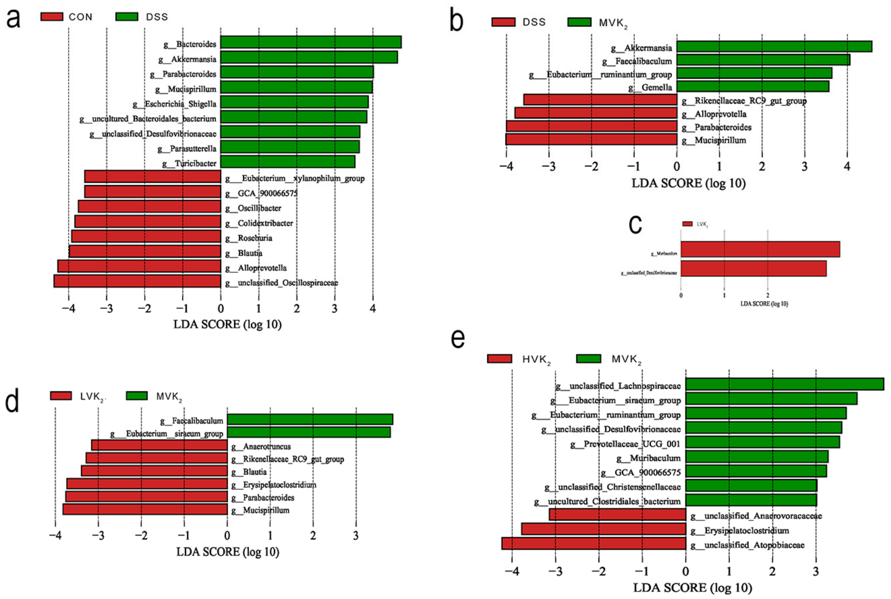

Figure S1. LEFSe analysis. Discriminative functional pathway abundant between, CON versus DSS (**a**), DSS versus MVK2 (**b**), LVK2 versus HVK2 group (**c**), LVK2 versus MVK2 (**d**), and MVK2 versus HVK2 (**e**).

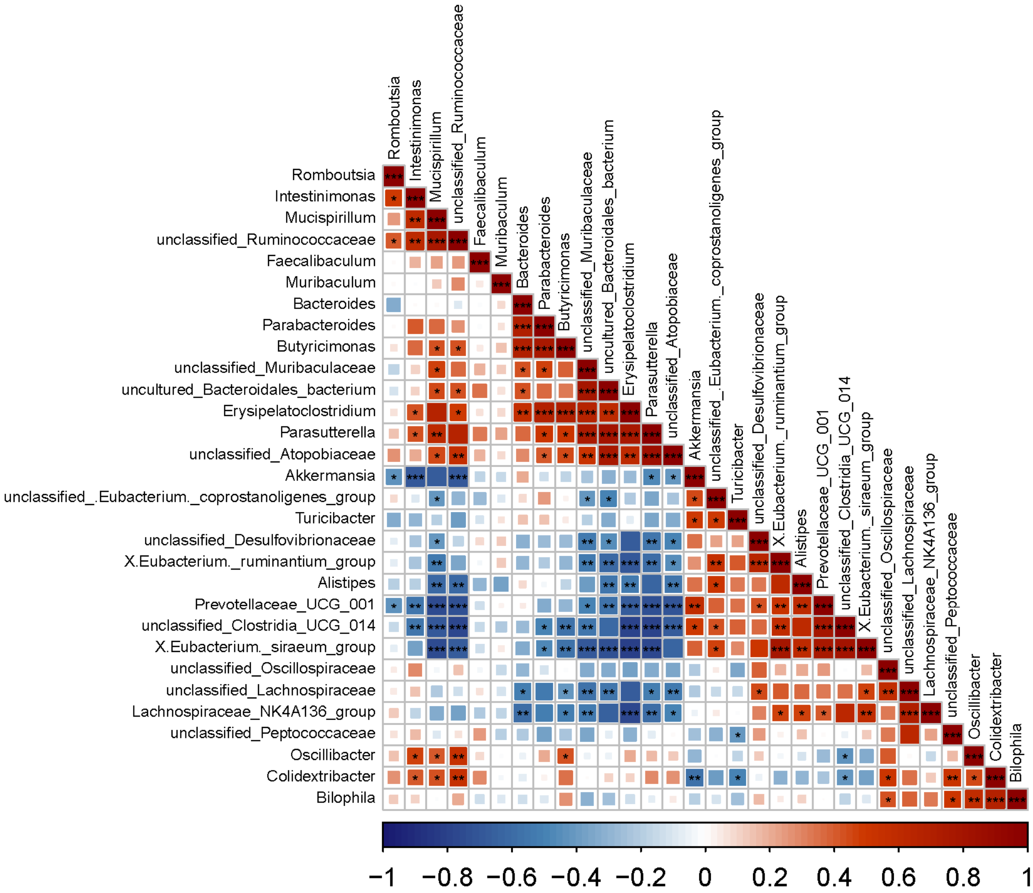

Figure S2. Positive and negative correlation matrix between the top 30 most abundant bacterial taxa. Results of a pairwise Spearman's rank correlation after the final DSS administration are shown. Correlations with P values less than 0.05 are marked with asterisk symbols and adjusted by the Benjamini-Hochberg FDR method. Related genera based on Euclidean distance were clustered together. Red, positive correlation; blue, negative correlation.

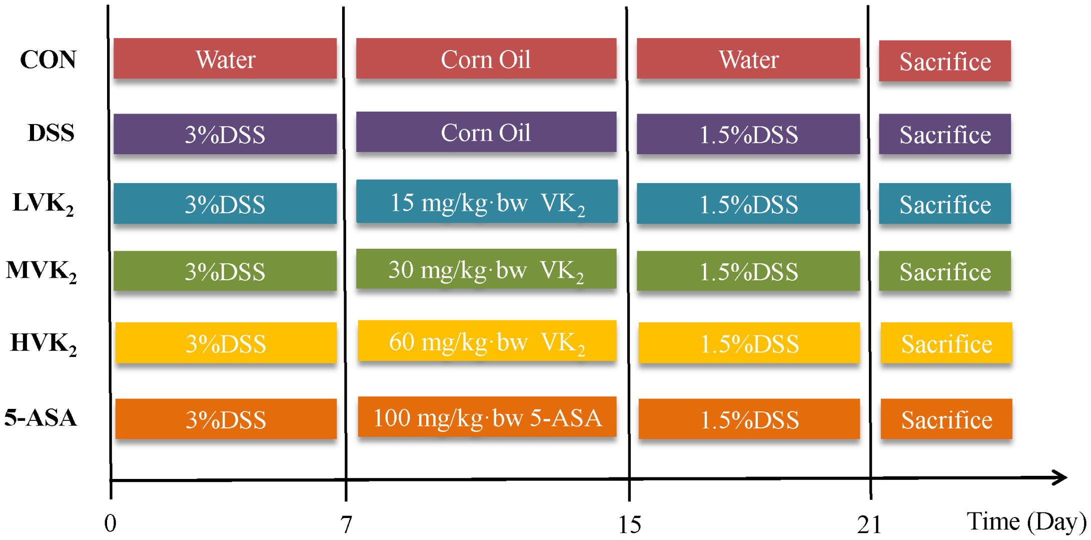

Figure S3. Scheme of the treatments. Except for the CON group, the other groups received a 3% DSS solution for molding on days 0-7. Corn oil gavage was administered to the CON and DSS groups on days 8–21 of the trial, and various doses of VK2 and 5-ASA were given to the LVK2, MVK2, HVK2, and 5-ASA groups, respectively. On days 15-21 of the experiment, except for the CON group, the remaining groups were again quoted 1.5% DSS solution for maintaining the model state.

Table S1. Scoring system for Disease Activity Index.

Score	Weight Loss	Feces Appearance	Blood in Feces
0	0%	Normal	No blood
1	1–5%	Loose	
2	5–10%	Watery diarrhea	Observed blood
3	10–20%	Slimy diarrhea, little blood	
4	>20%	Severe watery diarrhea with blood	Gross bleeding
